# Does rTMS Alter Neurocognitive Functioning in Patients with Panic Disorder/Agoraphobia? An fNIRS-Based Investigation of Prefrontal Activation during a Cognitive Task and Its Modulation via Sham-Controlled rTMS

**DOI:** 10.1155/2014/542526

**Published:** 2014-03-18

**Authors:** Saskia Deppermann, Nadja Vennewald, Julia Diemer, Stephanie Sickinger, Florian B. Haeussinger, Swantje Notzon, Inga Laeger, Volker Arolt, Ann-Christine Ehlis, Peter Zwanzger, Andreas J. Fallgatter

**Affiliations:** ^1^Department of Psychiatry and Psychotherapy, University of Tuebingen, Calwerstr 14, 72076 Tuebingen, Germany; ^2^Mood and Anxiety Disorders Research Unit, Department of Psychiatry and Psychotherapy, University of Muenster, Albert-Schweitzer-Campus 1, Building A9, 48149 Muenster, Germany; ^3^Department of Clinical Psychology and Psychotherapy, Universitaetsstr 31, 93053 Regensburg, Germany; ^4^kbo-Inn-Salzach-Hospital, Gabersee 7, 83512 Wasserburg am Inn, Germany; ^5^Graduate School LEAD, University of Tuebingen, Europastr. 6, 72072 Tuebingen, Germany; ^6^Cluster of Excellence CIN, University of Tuebingen, Otfried-Mueller-Str. 25, 72076 Tuebingen, Germany

## Abstract

*Objectives*. Neurobiologically, panic disorder (PD) is supposed to be characterised by cerebral hypofrontality. Via functional near-infrared spectroscopy (fNIRS), we investigated whether prefrontal hypoactivity during cognitive tasks in PD-patients compared to healthy controls (HC) could be replicated. As intermittent theta burst stimulation (iTBS) modulates cortical activity, we furthermore investigated its ability to normalise prefrontal activation. *Methods*. Forty-four PD-patients, randomised to sham or verum group, received 15 iTBS-sessions above the left dorsolateral prefrontal cortex (DLPFC) in addition to psychoeducation. Before first and after last iTBS-treatment, cortical activity during a verbal fluency task was assessed via fNIRS and compared to the results of 23 HC. *Results*. At baseline, PD-patients showed hypofrontality including the DLPFC, which differed significantly from activation patterns of HC. However, verum iTBS did not augment prefrontal fNIRS activation. Solely after sham iTBS, a significant increase of measured fNIRS activation in the left inferior frontal gyrus (IFG) during the phonological task was found. *Conclusion*. Our results support findings that PD is characterised by prefrontal hypoactivation during cognitive performance. However, verum iTBS as an “add-on” to psychoeducation did not augment prefrontal activity. Instead we only found increased fNIRS activation in the left IFG after sham iTBS application. Possible reasons including task-related psychophysiological arousal are discussed.

## 1. Introduction

According to DSM-IV, panic disorder (PD) is characterised by the sudden onset of unexpected panic attacks resulting in constant worries about possible reasons and negative consequences of the attacks. Moreover, in the case of comorbid agoraphobia, this eventually leads to behavioural avoidance of situations from which escape might be difficult in case of an attack [1]. On a neurobiological level, functional imaging studies of PD-patients with and without agoraphobia have found hypoactivity of the prefrontal cortex (PFC), paired with hyperactivity of fear relevant brain structures such as the amygdala, suggesting an inadequate inhibition by the PFC in response to anxiety-related stimuli [2–4]. In fact, hypofrontality of PD-patients has not just been observed in response to emotional stimuli [5], but also during cognitive tasks without any emotional content. For example, in a near-infrared spectroscopy study, Nishimura et al. [6] reported hypoactivation of the left PFC in particular while Ohta et al. [7] found that PD-patients as well as patients with a depressive disorder showed lower bilateral prefrontal activation than healthy controls during a verbal fluency task. Moreover, Nishimura et al. [8] investigated a potential relation between the frequency of panic attacks/agoraphobic avoidance and PFC activation during a cognitive task, indeed finding an association between altered activation patterns in the left inferior prefrontal cortex and panic attacks as well as between the anterior part of the right PFC and the severity of agoraphobic avoidance.

Cortical activation patterns can be selectively modified by means of repetitive transcranial magnetic stimulation (rTMS) via electromagnetic induction [9]. This way, rTMS has been shown to modulate neurotransmitter release [10] and—depending on its stimulation frequency—normalise prefrontal hypoactivity [11]. In fact, even though results are still inconsistent [12], rTMS has been shown to have a moderate antidepressant effect [13, 14]. Within this framework it is of special interest that the method does not just seem to alter affective states but also cognitive functioning [15, 16].

Functional near-infrared spectroscopy (fNIRS) is an imaging method which allows for a less complicated and faster application compared to other imaging methods such as functional magnetic resonance imaging (fMRI) or positron emission tomography (PET) [17]. Especially psychiatric patients with claustrophobic fears benefit from the fact that they merely need to sit in a chair while optodes that emit and receive near-infrared light are attached to their heads [18]. This way, task-related changes in oxygenated and deoxygenated haemoglobin concentrations can be examined. Even though disadvantages such as a relatively low spatial resolution (approximately 3 cm), a limited penetration depth (approximately 2 to 3 cm) [19, 20], and influences of extracranial signals do exist (for a review see [21]), fNIRS has proven to be a useful tool in psychiatric research [22].

Based on these findings and considerations, the goal of the current study was to (1) clarify whether the findings of Ohta et al. [7] concerning prefrontal hypoactivity in PD-patients compared to healthy controls during a cognitive paradigm (verbal fluency task) could be replicated via fNIRS in a larger sample. Also, a sham-controlled rTMS protocol was applied over the time course of three weeks above the left DLPFC to (2) examine whether excitatory rTMS can serve as an adequate tool in order to improve cognitive dysfunction in terms of prefrontal hypoactivation in PD-patients. In this regard, the patients' behavioural performance during the verbal fluency task was also taken into account.

## 2. Materials and Methods

### 2.1. Participants

Patients were recruited via the outpatient departments of the two study centres, advertisement in newspapers, as well as the internet and information material sent to local physicians. Exclusion criteria for all participants were age under 18 and over 65 years, pregnancy, and severe somatic disorders (e.g., cardiovascular disease, epilepsy, and neurological disorders). Also, patients fulfilling rTMS contraindications such as ferromagnetic implants or significant abnormalities in routine EEG were excluded. All patients were diagnosed with PD with or without agoraphobia according to DSM-IV-TR criteria [1]. Nonprominent comorbid psychiatric disorders (except for bipolar or psychotic disorder, borderline personality disorder, acute substance abuse disorders, and acute suicidality) were no exclusion criteria. Psychopharmacological treatment was permitted if the dosage had been stable for at least three weeks prior to baseline assessment (*t*1). Benzodiazepines, tricyclic antidepressants (except for Opipramol), and antipsychotics (expect for Quetiapine with maximal dosage of 50 mg) were excluded. Healthy controls who suffered from any axis-I psychiatric disorder (except for specific phobia) or had a family history of psychiatric disorders were excluded. A total of 23 controls and 44 PD-patients, of which 22 were randomised to the sham and 22 to the verum rTMS group, were selected for the study. Groups did not differ with respect to gender, age, years of education, and handedness (Table 1). After a comprehensive study description, written informed consent was obtained. The study was approved by the Ethics Committees of the Universities of Muenster and Tuebingen and all procedures were in accordance with the latest version of the Declaration of Helsinki.

### 2.2. Design

PD-patients received a total of 15 rTMS applications during three weeks at one of the study centres (Muenster or Tuebingen). Before the first and after the last rTMS-session brain activation was assessed with fNIRS while patients were performing a cognitive task. Between the first and the second fNIRS assessment, all patients received three group sessions of psychoeducation concerning PD. Healthy control subjects attended the two fNIRS measurements but received no rTMS in-between. Enrolment took place between January 2011 and July 2013. Patients and therapists were blinded to rTMS group assignment. This investigation was conducted within the framework of a larger study which included 9 weeks of cognitive behavioral therapy for patients with panic disorder/agoraphobia and additional fNIRS investigations described elsewhere (Deppermann et al., in preparation [23]).

### 2.3. Psychoeducation

Psychoeducation sessions were held in groups of up to 6 participants and were conducted by trained psychologists, who were supervised regularly by clinical psychotherapists. A state-of-the-art, standardised treatment manual was used [24, 25]. The content of the sessions included information about the pathogenesis of PD and agoraphobia, the vicious cycle of anxiety, somatic components of anxiety, and the sharing of personal experiences among the patients.

### 2.4. Verbal Fluency Task (VFT)

All subjects were assessed twice within a three-week interval between the first (*t*1) and the second (*t*2) measuring time.

During the measurements participants sat in a comfortable chair and were advised to keep their eyes closed and relax in order to avoid head or body movements. The VFT consisted of a phonological, a semantical and a control task. During the phonological task, subjects were instructed to produce as many nouns as possible beginning with a certain letter, whereas during the semantical task they had to name as many nouns as possible belonging to a certain category while repetitions and proper nouns were supposed to be avoided. During the control task the participants were instructed to repeat the weekdays in a speed that approximately matched the number of recited days to the number of mentioned nouns. The VFT started with a resting state phase of 10 seconds followed by the different tasks and more resting state periods, which lasted 30 seconds each. The sequence of the three tasks and resting phases were repeated three times, each time with a different letter or category. The letters and categories were chosen from the “Regensburger Wortflüssigkeitstest” [26]. Different letters/categories were used at *t*1 and *t*2 and counterbalanced between subjects. During the resting phase, participants were told to relax.

### 2.5. rTMS

Starting after the first fNIRS measurement, intermittent theta burst stimulation (iTBS, [27]) was applied in the patient group during 15 daily sessions on workdays during three weeks with a figure-of-eight coil (MCF-B65, 2 × 75 mm diameter, *n* = 34, MAGSTIM 9925-00, 2 × 70 mm, *n* = 9) by means of a MagOption/MagPro X100 stimulator (MagVenture, Denmark, *n* = 34) and a MAGSTIM RAPID^2^ T/N 3567-23-02 stimulator (*n* = 9), respectively. ITBS was used in order to achieve a facilitating effect on cortex excitability, as this could be demonstrated for the motor cortex, but also for more frontal cortex areas in previous studies [27, 28]. The iTBS protocol consisted of a total of 600 pulses applied in intermittent biphasic bursts at a frequency of 15 pulses per second via 2 second trains, starting every 10 seconds as described by Huang et al. [27]. The time of day for iTBS application did not vary for more than 2 hours from one day to the next. As the circadian rhythm is known to influence cortical excitability [29] the participants' individual resting motor threshold was determined prior to each iTBS session on the left motor cortex and stimulation intensity was set to 80% of this threshold. Stimulation site was F3 (left DLPFC) according to the international 10–20 system for electrode placement [30]. In order to ensure that the site of stimulation stayed constant over all sessions, F3 was drawn onto an individual textile cap for each participant prior to the first session. Additionally, other orientation points as the nasion, the inion, and the auricles were sketched on. While the coil was held tangentially to the scalp forming a 45° angle to the mid-sagittal line of the head (handling pointing in a posterior direction) for verum stimulation, it was flipped away from the scalp in a 90° angle for the sham stimulation. The post-fNIRS measurement (*t*2) was set to be conducted no earlier than 12 hours after the last rTMS-session to avoid the measurement of* acute* rTMS effects.

### 2.6. fNIRS

Relative temporal changes in oxygenated (O_2_Hb) and deoxygenated haemoglobin (HHb) were measured from a 10-second baseline using the ETG-4000 optical topography system (Hitachi Medical Co., Japan). For this purpose, the ETG-4000 uses laser diodes which emit light of two wavelengths (695 ± 20 nm and 830 ± 20 nm) and photodetectors which receive the scattered light intensity. Since the main light absorbers in this setup are the two types of haemoglobin, changes in measured light intensity between the emitter-detector pairs can be related to haemodynamic changes—which are coupled to neural activation—using a modified Beer-Lambert equation [31]. Altogether the probe set consisted of 16 photodetectors and 17 light emitters arranged in a 3 × 11 fashion with an interoptode distance of 3 cm resulting in 52 distinctive channels with a penetration depth of approximately 2 to 3 cm [19, 20]. The probe set was attached over the participants' prefrontal cortex having the central optode of the lowest row on FPz stretching out towards T3 and T4, respectively, according to the 10–20 international EEG system [32]. The sampling frequency was 10 Hz. The unit used to quantify haemoglobin concentration changes was mmol × mm. Subsequently, the recorded data were averaged over the corresponding blocks and exported into Matlab R2012b (The Math Works Inc., Natick, USA) where they were first corrected for changes in the NIRS signal that were not directly due to functional changes in haemoglobin concentration related to the attended tasks. To this end, frequencies that exceeded 0.05 Hz were removed using a low pass filter and clear technical artefacts (e.g., due to an optode losing contact to the scalp during measurement) were corrected by means of interpolation by replacing the values of the corresponding channels with the values of the circumjacent channels in a Gaussian manner (closer channels were taken more into account). In order to further remove artefacts, due to head movements, a correlation-based signal improvement (CBSI) procedure according to Cui et al. [33] was applied, adjusting the values for each channel by the equation

 
$[\text{CBSI}]\;=\;0.5*\left(\left[{\text{O}}_{2}\text{Hb}\right]-\frac{\text{std}\left[{\text{O}}_{2}\text{Hb}\right]}{\text{std}\;\left[\text{HHb}\right]*\left[\text{HHb}\right]}\right)$.

According to this approach, cortical activation should result in a negative correlation between O_2_Hb and HHb concentrations so in case of positive correlations the O_2_Hb signal is adjusted. Even though exceptions regarding a strictly negative correlation during brain activation exist [34], Brigadoi et al. [35] showed promising results for this procedure. Finally, the CBSI adjusted signal was once more interpolated in a Gaussian manner by using an inner-subject variance threshold of 4 as an interpolation criterion, assuming that exceeding values were most likely the result of further artefacts. Altogether a total of 5% of all channels were replaced.

After preprocessing, the data were averaged for all three groups within a time frame of 0–45 seconds after the onset of each task. The amplitude integrals in CBSI concentration between 5 and 40 seconds were taken as the basis for statistical analysis as a delay of the haemodynamic response after task onset can be assumed.

### 2.7. Regions of Interest (ROI)

**Figure 1:**
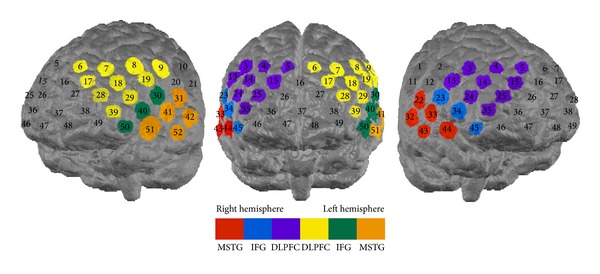
Probe set arrangement with numbers indicating channels. DLPFC: dorsolateral prefrontal cortex, IFG: inferior frontal gyrus, MSTG: middle superior temporal gyrus, color-coded channels were used for analyses.

Based on prior studies investigating verbal fluency [6–8, 36, 37], different a priori ROIs were defined. Accordingly, in addition to temporal areas (middle and superior temporal gyrus (MSTG)) and the inferior frontal gyrus (IFG) comprising Broca's area, the DLPFC is also supposed to be critically involved when performing a VFT. Corresponding channels were chosen using a virtual registration procedure as described by Tsuzuki et al. [38], Rorden and Brett [39], and Lancaster et al. [40] (cf.  Figure 1).

### 2.8. Clinical Assessment

PD with or without agoraphobia was diagnosed by experienced clinical psychologists with the German version of the Structured Clinical Interview for DSM-IV, Axis I Disorders (SCID-I [41, 42]). Anxiety was measured with the following questionnaires:* Panic and Agoraphobia Scale* (PAS; [43]),* Hamilton Anxiety Rating Scale* (HAM-A; [44]), and* Cardiac Anxiety Questionnaire* (CAQ; [45, 46]). All questionnaires were completed at *t*1 and *t*2. For all scales, higher scores indicate more severe symptoms.

In case of missing questionnaire items, a last observation carried forward analysis (LOCF) was conducted. If less than 10% of all items were left out, missing values were substituted by the participant's mean on the relevant scale.

### 2.9. Statistical Analyses

All analyses were conducted with IBM SPSS Statistics 20 and 21, respectively. The sample characteristics were assessed by means of *χ* ^2^ tests (gender, handedness, and first language) or *t*-tests (age, years of education, duration of illness for patients, and questionnaire data for *t*1 and *t*2), directly comparing the experimental groups (active versus sham, sham versus controls, and active versus controls). If numbers for the corresponding categories were below 5, Fisher's exact test was considered instead of asymptotic significance. The effects of patients' blinding regarding rTMS treatment condition were evaluated using binomial tests (test proportion: 0.5) for the subjectively perceived rTMS condition in each patient group, separately. The optimal sample size was determined based on previous studies investigating the effect of high-frequency rTMS on symptom severity in depression (e.g., [47]). The effect size of such a treatment protocol was estimated to approximate 0.5, while power was defined as 80%. The *α*-level was set to 5%. Since the effect of rTMS protocols in patients suffering from anxiety disorders is still difficult to quantify [48], it was decided to follow a more conservative assessment resulting in a target sample size of *n* = 40 patients.

For baseline assessment, fNIRS-data for all ROIs were analysed by means of analyses of variance (ANOVA) with the between-subject factor group (patients versus controls). The corresponding behavioural performance was analysed accordingly. In order to verify that changes in CBSI concentration were task-related, effects of hemispheric lateralisation were further analysed using a 2 × 3 repeated measurement ANOVA (RM-ANOVA) with the within-subject factors hemisphere (left versus right) and task (semantical versus phonological versus control task). As the factor time was of no relevance within this context, the corresponding data were averaged across the two measurement times. Accordingly, the phonological and semantical task should elicit a left lateralisation in the language relevant ROIs (IFG & MSTG) [36].

To evaluate the effects of rTMS on prefrontal activity, 2 × 3 RM-ANOVAs for each ROI and cognitive task were conducted (within-subject factor time (*t*1 versus *t*2), between-subject factor group (verum versus sham versus controls)).

The total number of produced nouns for the phonological and semantical task was investigated according to the collected fNIRS-data via a 2 × 3 RM-ANOVA with the within-subject factors time (*t*1 versus *t*2) and the between-subject factor group (verum versus sham versus controls). The number of weekdays was not considered in the analysis as it was matched to fit the number of nouns in the other tasks.

In case of violations of the sphericity assumption, the degrees of freedom in the ANOVAs were corrected using the Greenhouse-Geisser or Huynh-Feldt procedure depending on *ε* (*ε* > 0.75 Huynh-Feldt, *ε* < 0.75 Greenhouse-Geisser; see [49]). To avoid *α*-error accumulation due to multiple testing, the significance level of *α* = 0.05 was adjusted using a Bonferroni-Holm (BH) [50] correction procedure for the ROIs in each hemisphere, separately. Post hoc analysis was conducted by means of two-tailed *t*-tests for paired and independent samples.

In order to assess the relationship between cortical activation and behavioural performance, correlations between the number of recited words and CBSI-concentration were calculated at *t*1 and *t*2 for each group and task separately by means of Spearman's rho. To further directly consider changes over time, correlations between the differences (*t*2−*t*1) in CBSI concentrations and number of recited words were calculated. For post hoc *t*-tests and correlations, one-tailed *P*-values were considered in case of directed hypotheses.

## 3. Results

### 3.1. Sample Characteristics

Tables 1 and 2 give an overview of the sociodemographic sample characteristics at baseline and clinical questionnaire data for *t*1 and *t*2. Sociodemographic data did not differ between groups. For the clinical questionnaire data, no significant differences emerged between the sham and verum stimulated group for *t*1. Verum group versus controls and sham group versus controls, respectively, revealed significant differences on all scales in the expected directions (data shown for HAM-A, self-rated PAS, and CAQ, Table 2).

When patients were asked to guess whether they had received active or sham rTMS, 16 patients in the sham group thought that they had been sham stimulated while 5 thought that it had been the active protocol. Fourteen patients in the verum group thought they had obtained the active protocol and 4 said that they received a placebo treatment. Additionally, 5 patients (1 sham, 4 verum) did not reply to the question. For each patient group, these guesses differed significantly from chance (binomial test, sham group: *P* = 0.027 and verum group: *P* = 0.031).

### 3.2. Behavioural Performance

Table 3 contains means and standard deviations for the number of produced nouns for the phonological as well as the semantical task for each group and each measuring time.

With respect to behavioural data, no significant baseline differences could be found between patients and controls. Further the 2 × 3 RM-ANOVA revealed no significant changes for either the phonological or the semantical task.

### 3.3. Prefrontal Activity at Baseline

**Figure 2:**
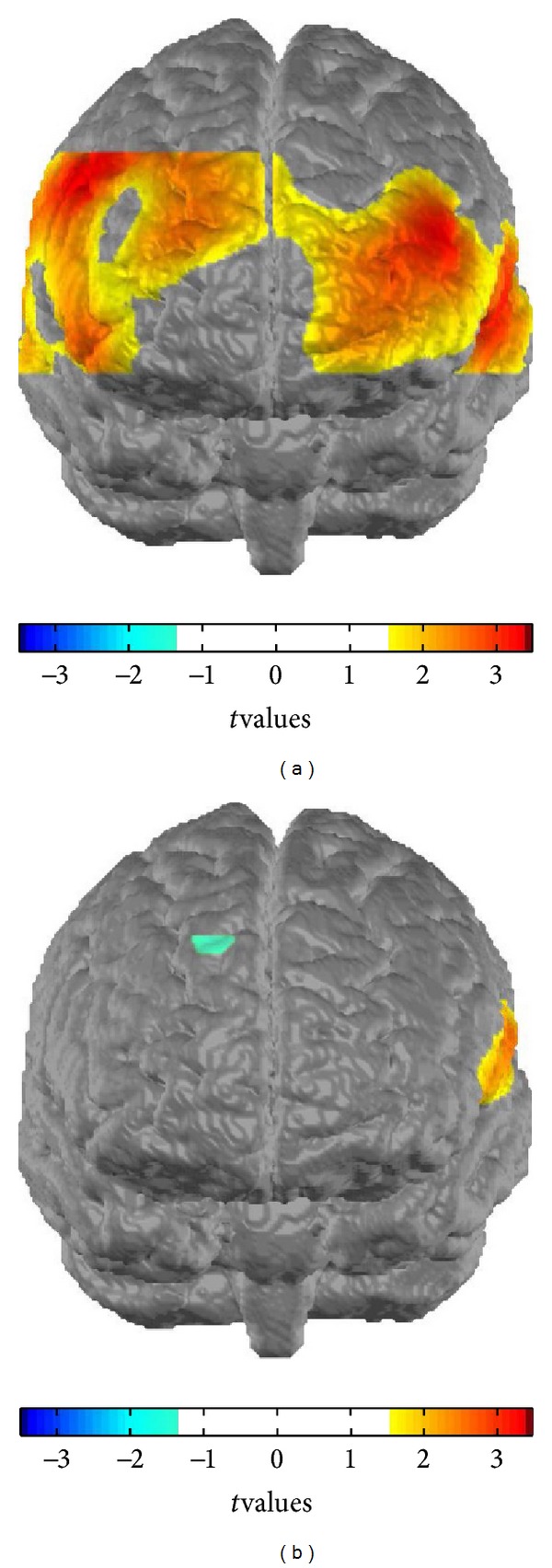
Contrast maps phonological task. Differential CBSI concentration levels contrasted between groups ((a) controls versus PD-patients and (b) verum versus sham) for the phonological task at baseline. Differences in CBSI levels between groups are depicted by means of *t*-values for each channel, whereby only values for *t* ≥ 1.7 are shown.

**Figure 3:**
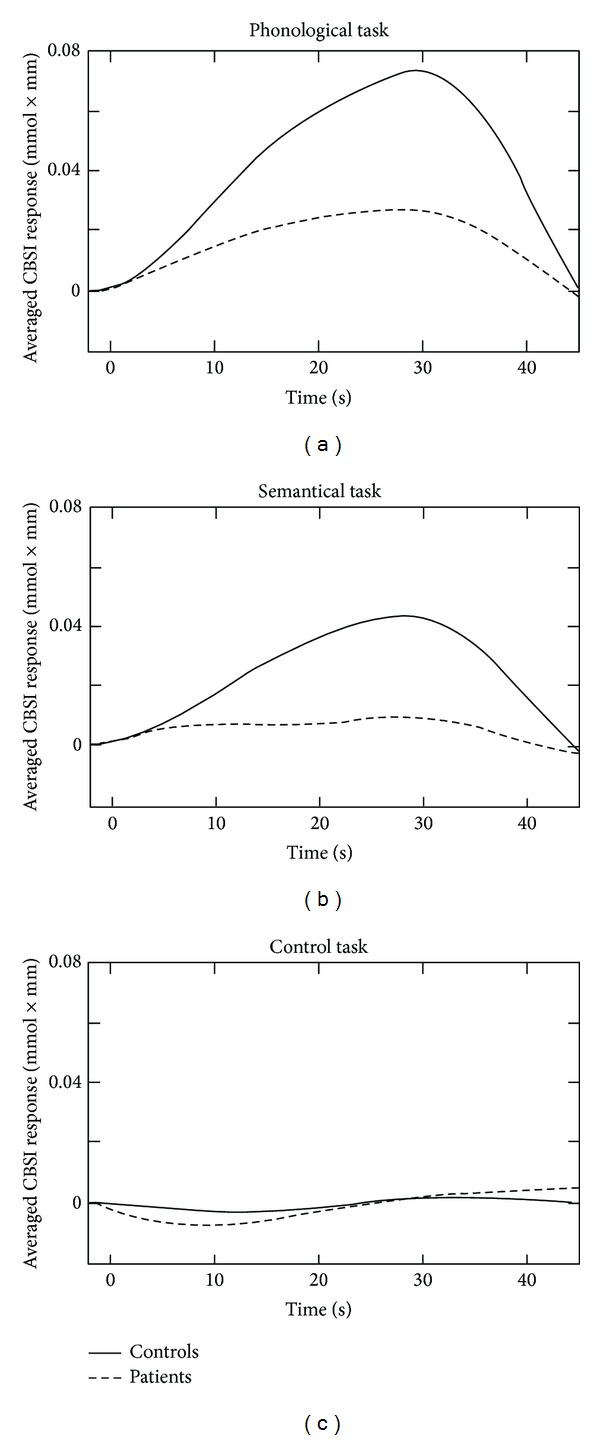
Haemodynamic response function of the left dorsolateral prefrontal cortex at the baseline measurement, averaged over all subjects for each task, separately.

Because one patient missed *t*2, the fNIRS-data of this subject were excluded from all analyses. Concerning the remaining subjects, significant results were found for all ROIs on both hemispheres for the phonological task (Figure 2) whereby the healthy controls displayed more activation than the patients (left DLPFC: *F*_1,65_ = 9.304, *P* = 0.003, left MSTG: *F*_1,65_ = 8.795, *P* = 0.004, left IFG: *F*_1,65_ = 5.279, *P* = 0.025, right DLPFC: *F*_1,65_ = 11.649, *P* = 0.001, right MSTG: *F*_1,65_ = 5.158, *P* = 0.026, right IFG: *F*_1,65_ = 8.130, *P* = 0.006, all *P* BH-corrected). For the semantical task significant differences in terms of higher activation in the healthy controls were found only for the DLPFC bilaterally (left DLPFC: *F*_1,65_ = 6.189, *P* = 0.015, right DLPFC: *F*_1,65_ = 11.176, *P* = 0.001, all *P* BH-corrected). For the control task no significant differences were found (Figure 3).

### 3.4. Effects of Hemispheric Lateralisation

Regarding hemispheric lateralisation effects, the 2 × 3 RM-ANOVA showed a significant main effect for the two language related ROIs IFG (*F*_1,65_ = 15.030, *P* < 0.001 (<0.0167, BH-corrected)) and MSTG (*F*_1,65_ = 8.317, *P* = 0.005 (<0.025, BH-corrected)) where activation—as indicated by CBSI concentration—was higher for the left hemisphere. A significant main effect of task was identified for all ROIs (DLPFC: *F*_2,100_ = 24.275*P* < 0.001 (<0.0167, BH-corrected), MSTG: *F*_2,100_ = 55.974*P* < 0.001 (<0.025, BH-corrected), and IFG: *F*_2,100_ = 61,718*P* < 0.001 (<0.05, BH-corrected)). The interaction hemisphere∗task was significant for the IFG (*F*_2,130_ = 8.151, *P* < 0.001 (<0.0167, BH-corrected) and the MSTG (*F*_2,114_ = 3.478, *P* = 0.040 (<0.05, BH-corrected)). Post hoc analyses showed that this was due to a left lateralisation concerning the phonological (IFG, right versus left: *t*_65_ = −3.734, *P* < 0.001 and MSTG, right versus left: *t*_65_ = −2.983, *P* = 0.002) and partly the semantical (IFG, right versus left: *t*65 = −4.034, *P* < 0.001) task while there was no significant difference for the control task. Regarding the DLPFC, no significant main effect of hemisphere was found, whereas the interaction hemisphere∗task was significant (*F*_2,130_ = 11.040, *P* < 0.001 (<0.025, BH-corrected)). For the DLPFC, results were in contrast to the above-mentioned findings with a significant lateralisation effect in terms of increased activation in the right hemisphere for the control task (*t*_65_ = 5.072, *P* < 0.001) but no significant difference for the two active verbal fluency tasks. Differences between tasks were significant for all comparisons for the IFG (right hemisphere: *t*_65_ ≥ 2.7, *P* ≤ 0.005 and left hemisphere: *t*_65_ ≥ 3.37, *P* < 0.001) and left MSTG (*t*_65_ ≥ 3.322, *P* < 0.001) with activation during the phonological task > activation during the semantical task > the control task. For the right hemisphere of the DLPFC, activation during the phonological task was also higher than for the semantical task (*t*_65_ = 6.083, *P* < 0.001). For the left DLPFC, participants showed similar activation patterns as for the IFG and left MSTG with respect to the three test tasks (phonological > semantical > control, for all: *t*_65_ ≥ 3.114, *P* ≤ 0.0015).

### 3.5. Effects of rTMS on Prefrontal Activity

For the left DLPFC, the analyses of the phonological task showed a significant main effect of group (*F*_2_, 63 = 5.32, *P* = 0.007 (<0.0167, BH-corrected)). Post hoc analyses revealed that this was due to significantly lower cortical activation of patients in the sham (*t*_42_ = −2.13, *P* = 0.02) and verum group (*t*_43_ = −2.74, *P* = 0.005) compared to healthy controls. No significant interaction effect of time and group or main effect of time was found. For the right DLPFC, a significant main effect of group (*F*_2,63_ = 5.34, *P* = 0.007 (<0.0167, BH-corrected)) was found. No significant effect of time or significant interaction effect of time and group existed with respect to the phonological task. Post hoc *t*-tests displayed similar results as for the left DLPFC. Verum and sham stimulated patients showed a reduced activation compared to healthy controls (for both: *t*_32_ ≤ −2.348, *P* ≤ 0.013).

For the semantical task, a significant main effect of group was found for the left and the right DLPFC (for both: *F*_2,63_ ≥ 5.30, *P* ≤ 0.007 (<0.0167, BH-corrected)). For both areas, actively stimulated patients showed a significantly reduced cortical activation compared to healthy controls (left DLPFC: *t*_35_ = −2.78, *P* = 0.005 and right DLPFC: *t*_43_ = −2.60, *P* = 0.007). Also, sham stimulated patients showed significant hypoactivation compared to healthy participants with respect to the right (*t*_38_ = −3.19, *P* = 0.002) and left DLPFC (*t*_34_ = −2.316, *P* = 0.014). No significant main effects of time or significant interactions of time and group were discerned for the left and right DLPFC, respectively. No significant differences between sham and verum stimulated patients existed with regard to the left or right DLPFC for the phonological and semantical task, respectively.

The analyses of the control task for the left and right DLPFC revealed neither significant main effects of group nor significant main effects of time. Also, no significant interaction effects of time and group were found.

For reasons of clarity, solely significant results for the IFG with respect to the three test tasks are depicted in Table 4. For the MSTG, no significant outcomes were found.

### 3.6. Correlations between fNIRS Data and Behavioural Performance

At baseline, no significant correlations between CBSI concentration and the number of recited words were found for either PD-patients or for the healthy controls. At the second measurement time, a relationship was merely found for the healthy controls in terms of negative correlations for all ROIs, except for the right DLPFC with the number of recited words during the phonological task (left DLPFC: *r* = −0.416, *P* = 0.024, left MSTG: *r* = −0.431, *P* = 0.020, left IFG: *r* = −0.452, *P* = 0.015, right MSTG: *r* = −0.534, *P* = 0.004, right IFG: *r* = −0.558, *P* = 0.003, all *P* BH-corrected). Regarding changes over time, significant results existed only during the phonological task in the two patients' groups. In this context, an increase in the number of recited words was significantly associated with a decrease in CBSI concentration (resp., vice versa) for the DLPFC (sham, left DLPFC: *r* = −0.498, *P* = 0.011, verum, left DLPFC: *r* = −0.485, *P* = 0.011, verum, right DLPFC: *r* = −0.607, *P* = 0.001, all *P* BH-corrected). As all correlations were negative, they were only considered explorative, as positive correlations were hypothesized and one-sided tests were conducted.

## 4. Discussion

The present study aimed to confirm the finding that PD-patients are characterised by prefrontal hypoactivation during cognitive tasks as compared to healthy controls [7]. Moreover, it additionally addressed the question whether a potential hypoactivation of the PFC can be normalised by means of repeated iTBS. Patients with PD were investigated via fNIRS while performing a VFT prior to and after receiving daily prefrontal iTBS application over a time course of three weeks in addition to weekly group sessions of psychoeducation. The VFT-results were compared with those of healthy control subjects.

Regarding our first hypothesis, our results are in line with the above-mentioned findings concerning hypofrontality during cognitive tasks in PD-patients. With respect to our second hypothesis, unexpectedly, an increase in activation over time could only be found for the left IFG in sham stimulated patients.

In more detail, before the start of rTMS treatment, differences in cortical activation (as indicated by CBSI data) between patients and controls were observed for specific task conditions of the VFT. In fact, as predicted by our hypothesis, patients did not differ from controls during the control task but displayed decreased prefrontal activation in all ROIs during the phonological task and partly also during the semantical task. The missing differences during the control task indicate that the differences in CBSI concentration between healthy controls and patients during the two active tasks were indeed due to altered cognitive processing and not to more general effects elicited by the measurement situation. Still, it cannot be excluded that our fNIRS signal may have been affected by components that are not directly related to cognitive processing but still lead to a (task-related) change in blood flow and hence a change of the measured signal. Regarding more general effects that might influence the fNIRS signal, a recent study by Takahashi et al. [51] showed that the verbal fluency task is particularly affected by confounding effects due to stress induced skin blood flow, especially for NIRS channels located over the forehead. In order to verify that we still mainly measured cortical activation, we presumed that lateralisation effects in terms of increased left hemispheric activation should be found for language related areas such as the MSTG and IFG but not for the DLPFC. Further, increases in these two ROIs should only exist for the semantical and phonological but not for the control task. In line with previous studies [36] we could confirm these assumptions and accordingly ascribe our finding mainly to differences in cortical activation.

Contrary to our second hypothesis, no significant changes in prefrontal activation after rTMS treatment could be found in the verum group. In fact, the only significant change was found for the sham group which showed an increase in CBSI concentration in the left IFG during the phonological task. As at first glance these findings are hard to interpret and we further analysed the prefrontal activation patterns in relation to the behavioural performance of healthy controls and the two patients groups.

When regarding only the behavioural data, descriptively, healthy controls could name more nouns than both patients groups; however, this difference was not significant. Further, when associating CBSI concentrations in the different ROIs with the number of recited nouns at baseline, no significant correlations could be revealed for either group. Interestingly, however, at the second measurement time, negative correlations between the behavioural performance and activation patterns in nearly all ROIs existed for the healthy controls. Even though we originally applied one-sided testing (assuming a positive relationship between behavioural performance and cortical activation), we still think that it is worthwhile to give these negative correlations some considerations as they might be helpful for a better understanding of our results.

Similar to the finding in healthy controls, negative associations between changes in the number of recited nouns from *t*1 to *t*2 and changes in DLPFC activation bilaterally during the phonological task could be found for both patients groups. In order to interpret these results in a meaningful way, it has to be considered that multiple distinct mechanisms might have an influence on the fNIRS signal. Firstly, according to our hypothesis, it can be assumed that a demanding cognitive task leads to an increase in cortical activation which then triggers a certain performance at the behavioural level. In this context, higher cortical activation should lead to a better behavioural performance as it implies that more cognitive resources can be recruited to fulfil the task as well as possible. From another perspective, one could also assume that in subjects with a highly efficient cortical processing (i.e., in case of a subjectively nonchallenging task situation) fewer cognitive resources are needed to achieve good results. In this case, low cortical activation should be associated with high behavioural performance. However, it needs to be kept in mind that the fNIRS signal might not just contain components which are due to cortical activation but might also be influenced by extracranial signal components that relate to peripheral processes such as psychophysiological arousal induced changes in blood flow. In particular, in frontopolar regions, these components have been shown to also trigger an increase in the fNIRS signal due to stress induced vasodilation during a verbal fluency task [51]. In this context, higher CBSI concentrations might then also be associated with a decrease in behavioural performance as it can be presumed that too much psychophysiological arousal should have a negative effect on cognitive functioning. Even though we tried to control for such arousal effects by performing a control task and considering lateralisation effects, we cannot exclude the fact that it still had an effect on our results.

Accordingly, we conclude that we could not find any significant correlations at the baseline measurement time as psychophysiological arousal was probably very high for all participants, hence having confounding effects on the fNIRS signal components due to cortical activation. At the second measurement time, cortical activation should have been the same for the healthy controls while arousal may have decreased for some participants as the situation was more familiar, leading to a reduction in signal intensity and negative correlations with behavioural performance due to improved cognitive function (with reduced arousal). While it cannot be excluded that these negative correlations also imply that the task was not challenging enough for some of the healthy subjects, the study by Takahashi et al. [51] points more in favour of an interpretation in terms of a decrease in psychophysiological arousal. In fact, the authors could show that already a repetition of the verbal fluency task within one measurement could lead to a significant repetition effect by means of a decrease in psychophysiological arousal and associated fNIRS signal intensity.

Concerning the PD-patients, psychophysiological arousal should have also decreased but possibly not as much as in the healthy controls as the measurement situation still represented a typical panic-relevant situation (patients had to sit in a small room with the fNIRS probe set attached to their heads so a sudden escape was not possible). At the same time it can be expected that arousal effects, which are prominent in the frontopolar area of the PFC, also have an effect especially on the DLPFC which cannot be neglected [52]. A possible explanation especially for the influence of DLPFC activation through the frontopolar region is given by Kirilina et al. [53] who found that the vein responsible for arousal effects in the forehead also stretches out to dorsolateral regions. Consequently, apparent effects of a slight decrease in arousal would most likely be expected in the DLPFC, hence explaining the negative correlations between changes in behavioural performance and changes in CBSI concentrations for the patients. Even though correlations between CBSI concentrations and behavioural performance during the semantical task were not significant, it is noteworthy to mention that the direction of the correlations was generally the same, supporting our prior assumptions.

We therefore conclude that healthy controls as well as patients in both groups were generally less affected by psychophysiological arousal during the second measurement time. In this regard, the increase in activation from the first to the second measurement time for the left IFG in the sham group might not be related to an increase in cognitive functioning but might merely represents a more general possibly also arousal related effect. A further reason which might have contributed to the increase in CBSI concentrations after sham iTBS might be given by simple regression towards the mean. In this regard it needs to be considered that sham and verum stimulated patients did not differ significantly in their activation patterns after rTMS application. Instead, sham stimulated patients showed a significantly decreased baseline CBSI concentration in the left IFG compared to healthy controls. All in all, our findings confirm our first hypothesis that PD-patients show a prefrontal dysfunction that is at least partly independent of panic-related tasks. However, an increase in cortical activation after verum iTBS was not found. Instead, we could accentuate the need to consider task-related arousal induced effects especially when investigating patients with anxiety disorders.

To our knowledge, this is the first controlled study investigating effects of add-on theta burst stimulation (TBS) on prefrontal activation and cognitive functioning in patients with PD/agoraphobia. So far, only a few open studies investigated the effects of TBS on psychiatric symptoms (e.g., [54, 55]).

However, limitations of this study have to be mentioned. The stimulation condition (verum versus sham) was correctly identified by the majority of patients, so one could argue that placebo effects might have affected our results. Possibly, patients exchanged their perceptions about rTMS during the psychotherapy group sessions, as they became acquainted with each other over the course of psychoeducation. For further investigations, we therefore emphasise the need for specialised sham coils which produce a superficial electrical current on the skull, as demonstrated by Rossi et al. [56]. Although in our study sufficient blinding could not be reached, promising results of rTMS in controlled studies with electromagnetic placebo coils could demonstrate specific effects of verum stimulation on psychiatric symptoms (e.g., for PTSD and comorbid depression by Boggio et al. [57]). Referring to the choice of the rTMS-frequency, we used a protocol which is assumed to facilitate motor cortex excitability [27]. Also, a facilitation of frontal activity could be demonstrated. For example, speech repetition accuracy was promoted by intermittent theta burst stimulation of the left posterior inferior frontal gyrus [28]. Nevertheless, rTMS effects seem to be influenced by a wide range of factors, for example, genetic variables or the way of application. Cheeran et al. [58] could demonstrate a significant influence of the brain-derived neurotrophic factor gene (BDNF) on the TBS-efficacy for the primary motor cortex. Also, TBS after-effects seem to hinge on the NMDA-receptor [59]. Further, a study of Gamboa et al. [60] demonstrated reversed iTBS-effects after a prolonged, single application of 1200 instead of 600 stimuli. Taken together, it could be questionable if iTBS consistently facilitates the excitability of stimulated neurons. Moreover, in our study, rTMS was generally applied after psychoeducation sessions. However, an application prior to psychoeducation could have led to a different processing of the afterwards presented information. We therefore suggest that future studies should systematically assess temporal effects of rTMS applications in relation to additional intervention methods. Regarding methodology, we have already discussed the problems that arise from the confounding skin blood flow signal component in the fNIRS data. A possible solution to this—which allows for an even more precise interpretation of the result—might be to measure the skin components selectively by additionally placing optodes with shorter interoptode distances on the probe set [51]. Finally, concerning the diagnostic process, PD/agoraphobia was diagnosed prior to *t*1 with the help of structured clinical interviews. However, the time lag between these interviews and *t*1 was not standardized in our study.

## 5. Conclusion

This pilot study investigated cortical activation patterns of patients with PD/agoraphobia compared to healthy controls. Further, effects of add-on iTBS on cortical activation and cognitive performance in PD/agoraphobia were analysed. Findings of a baseline cortical hypoactivation could be replicated. However, an increase in cortical activation after verum iTBS could not be supported. Instead we only found increased CBSI concentrations for the left IFG after sham iTBS application. By integrating behavioural performance into our analysis we could attribute this finding to more general effects such as task-related psychophysiological arousal and regression towards the mean. Taken together, our results confirm that PD is characterised by prefrontal hypoactivation. As we could not verify an increase in cortical activation after verum iTBS, further studies that should control for task-related psychophysiological arousal are needed in order to evaluate under which circumstances iTBS might serve as a therapeutic tool in the treatment of PD.

## Figures and Tables

**Table 1:** Sociodemographic sample characteristics.

	Age mean (range)	Gender	Handedness	First language	Years of education mean (SD)	Duration of illness in months mean (range)
Group						
Controls	33.4 (19–64)	14 females 9 males	20 right 3 left	22 German 1 bilingual	12.5 (1.1)	—
Sham	36.3 (22–56)	14 females 8 males	21 right 1 left	19 German 2 bilingual 1 other	12.4 (2.0)	84 (1–336)
Verum	37.6 (19–63)	13 females 9 males	20 right 2 left	19 German 1 bilingual 2 other	12.1 (1.7)	92 (1–372)
Comparisons						
Controls versus Sham	*t*_43_ = −0.921 *P* = 0.362	*χ*_1_^2^ = 0.037 *P* = 0.848	*χ*_1_^2^ = 1.003 *P* = 0.317	*χ*_2_^2^ = 1.531 *P* = 0.465	*t*_33_ = −0.234 *P* = 0.816	—
Controls versus Verum	*t*_43_ = −1.148 *P* = 0.257	*χ*_1_^2^ = 0.015 *P* = 0.903	*χ*_1_^2^ = 0.178 *P* = 0.673	*χ*_2_^2^ = 2.198 *P* = 0.333	*t*_37_ = −0.913 *P* = 0.367	—
Sham versus Verum	*t*_42_ = −0.399 *P* = 0.692	*χ*_1_^2^ = 0.096 *P* = 0.757	*χ*_1_^2^ = 0.358 *P* = 0.550	*χ*_2_^2^ = 0.667 *P* = 0.717	*t*_42_ = 0.490 *P* = 0.626	*t*_42_ = −0.290 *P* = 0.773

SD: standard deviation.

**Table 2:** Clinical characteristics of all groups, before and after rTMS treatment.

Group	*t*1 HAM-A mean (SD)	*t*2 HAM-A mean (SD)	*t*1 Self-rated PAS mean (SD)	*t*2 Self-rated PAS mean (SD)	*t*1 CAQ mean (SD)	*t*2 CAQ mean (SD)
Controls	3.83 (3.20)^a,b^	2.74 (3.57)^c,d^	0.22 (1.04)^a,b^	0.13 (0.34)^c,d^	0.33 (0.20)^a,b^	0.33 (0.22)^c,d^
Sham	20.3 (7.10)	15.20 (8.81)^e^	20.52 (8.10)	15.34 (8.30)^e^	1.36 (0.51)	1.06 (0.65)^f^
Verum	22.41 (8.97)	18.37 (10.05)^e^	20.76 (7.76)	14.91 (6.90)^f^	1.63 (0.71)	1.20 (0.71)^f^

Over the course of treatment, the degree of assessed symptoms on HAM-A, self-rated PAS, and CAQ significantly declined in the verum and sham stimulated group. However, no significant differences after rTMS-treatment between these two groups occurred. ^a^ *P* < 0.001 compared with sham rTMS (*t*1); ^b^ *P* < 0.001 compared with verum rTMS (*t*1); ^c^ *P* < 0.001 compared with sham rTMS (*t*2); ^d^ *P* < 0.001 compared with verum rTMS (*t*2); ^e^ *P* < 0.01  *t*-test for paired samples; ^f^ *P* < 0.001  *t*-test for paired samples; CAQ: cardiac anxiety questionnaire, HAM-A: Hamilton Anxiety Rating Scale, PAS: Panic and Agoraphobia Scale, rTMS: repetitive transcranial magnetic stimulation, SD: standard deviation, *t*1: measuring time 1, and *t*2: measuring time 2.

**Table 3:** Number of produced nouns for phonological and semantical task for *t*1 and *t*2.

Time	Controls		Sham		Verum	
	Phonological mean (SD)	Semantical mean (SD)	Phonological mean (SD)	Semantical mean (SD)	Phonological mean (SD)	Semantical mean (SD)
*t*1	20 (7.6)	37.2 (7.2)	18.4 (7.2)	33.2 (7.4)	16.9 (6.4)	34.3 (7.8)
*t*2	19.7 (7.0)	38.2 (10.1)	19.2 (7.2)	32.5 (7.4)	19.4 (7.8)	35.5 (8.8)

SD: standard deviation, *t*1: measuring time 1, and *t*2: measuring time 2 after 3 weeks.

**Table 4:** Significant results for the cognitive tasks with respect to the IFG.

ROI	df (df error)	*F*	*P*	Verum versus sham	Verum versus controls	Sham versus controls	Paired *t*-tests
Left IFG-phonological task							
Time × Group	2 (63)	5.23	0.008 (<0.0167, BH-corrected)	*t*1: ns. *t*2: ns.	*t*1: ns. *t*2: ns.	*t*1: S < HC** *t*2: ns.	S: *t*1 < *t*2* V: ns. HC: ns.

*significant at a significance level of ≤0.05, **significant at a significance level of ≤0.01, BH-corrected: Bonferroni-Holm-corrected, HC: healthy controls, IFG: inferior frontal gyrus, S: sham group, and V: verum group.
